# Groundwater Contamination by Uranium and Mercury at the Ridaura Aquifer (Girona, NE Spain)

**DOI:** 10.3390/toxics4030016

**Published:** 2016-08-16

**Authors:** Andrés Navarro, Xavier Font, Manuel Viladevall

**Affiliations:** 1 Department of Fluid Mechanics, School of Industrial and Aeronautical Engineering of Terrassa (ETSEIAT), Universidad Politècnica de Cataluña, Colón 7–11, Terrassa, Barcelona 08222, Spain; 2 Department of Geochemistry, Petrology and Geological Prospecting, University of Barcelona, Faculty of Geology, Zona Universitaria de Pedralbes, Barcelona 08028, Spain; xavierfont@ub.edu (X.F.); mviladevall@ub.edu (M.V.)

**Keywords:** uranium, mercury: groundwater, sediments, aquifer

## Abstract

Elevated concentrations of uranium and mercury have been detected in drinking water from public supply and agricultural wells in alluvial and granitic aquifers of the Ridaura basin located at Catalan Coastal Ranges (CCR). The samples showed high concentrations of U above the U.S. standards and the World Health Organization regulations which set a maximum value of 30 µg/L. Further, high mercury concentrations above the European Drinking Water Standards (1 μg/L) were found. Spatial distribution of U in groundwater and geochemical evolution of groundwater suggest that U levels appear to be highest in granitic areas where groundwater has long residence times and a significant salinity. The presence of high U concentrations in alluvial groundwater samples could be associated with hydraulic connection through fractures between the alluvial system and deep granite system. According to this model, oxidizing groundwater moving through fractures in the leucocratic/biotitic granite containing anomalous U contents are the most likely to acquire high levels of U. The distribution of Hg showed concentrations above 1 μg/L in 10 alluvial samples, mainly located near the limit of alluvial aquifer with igneous rocks, which suggests a possible migration of Hg from granitic materials. Also, some samples showed Hg concentrations comprised between 0.9 and 1.5 μg/L, from wells located in agricultural areas.

## 1. Introduction

Elevated concentrations of uranium (U) have been detected in drinking and mineral water in several countries through the world [1]. In that sense, for public water supplies, U.S. Standards [2] set a maximum value of 30 µg/L, and the World Health Organization proposed a maximum value of 30 µg/L [3], however, the European and Spanish legislation [4] does not consider U limitations. Uranium is the most abundant actinide element, reaching values of 2.2–15 ppm in granite [5] showing higher natural concentrations in groundwater related with granitic rocks, although rarely these values exceed 20 µg/L. Mean U crustal concentrations are comprised between 0.9 and 1.7 ppm, being somewhat higher in the case of acidic igneous rocks: 2.5–6 ppm [6]. Uranium can show different oxidation states: +4, +5 and +6, being the most abundant species in nature U (IV) and U (VI).

In USA aquifers, groundwater affected by the existence of U mineralizations showed concentrations between 1 and 120 µg/L, while in groundwater near U mines concentrations of 15–400 µg/L have been detected [5]. In other areas, with granitic lithology, high uranium concentrations were detected in groundwater, associated with igneous intrusions and contact metamorphic aureoles developed in schistose materials [7,8,9]. Besides, in granites and other igneous materials of the Norway crystalline bedrock, up to 18% of the groundwater samples showed concentrations above 20 µg/L [9]. Anomalies of U in groundwater and very high values of U in mine waters have also been detected in areas with U mineralizations associated with Cretaceous sediments, reaching concentrations up to 126 mg/L [10]. Furthermore, groundwater of sedimentary aquifers showed U values which reach 303.5 µg/L [11]. Also, the higher concentrations of U appear to be associated with mine waste leachates [12,13,14,15], showing dissolved U concentrations of <1 µg/L to 104 µg/L. In Germany, bottled mineral waters [16] showed U concentrations comprised between <0.0005 and 16.0 µg/L, while in Britain bottled waters [17] showed elevated concentrations of U associated with sandstone aquifers of Permotrias and Devonian age. In contrast, bottled waters from Norway, Sweden, Finland and Iceland showed high concentrations of U (29–32.4 µg/L) from wells located in granite and similar materials [18].

The mobility of U—as oxyanion is very sensitive to the redox conditions, being U (IV) the most stable state under reducing conditions and U (VI) in oxidizing conditions—showed in the first case a solubility lower than in the oxidized state. In this situation, the U mobilization may occur through the uranyl ion (UO_2_^2+^) at lower pH and their carbonate complexes at neutral and alkaline conditions. Thus, for pH > 5, the U (VI) appears generally as complex [UO_2_OH^+^, UO_2_(OH)_3_^−^] and more often as carbonate complex [(UO_2_)_2_CO_3_(OH)_3_^−^, UO_2_(CO_3_)_2_^2−^] [19]. The carbonate species are of great importance in U mobility, because it favors the solubility of U minerals, facilitates the oxidation of U (IV) and limits the sorption of U in oxidizing waters. Also, the formation of phosphate and fluoride complex favors the mobility of U.

The presence of U (VI) in natural waters may be associated with ore weathering of U (VI) mineral phases such as uraninite (UO_2_) and coffinite (USiO_4_), or the existence of autunite [Ca(UO_2_)_2_(PO_4_)_2_], which showed higher solubility [5]. Thus, contaminated groundwater modeled with PHREEQC code [20] showed supersaturation with respect to the uraninite, being UO_2.25_(U_4_O_9_) the phase which came closest to equilibrium with the concentrations of U detected in the water. The mobility of the U is also controlled by sorption processes and/or precipitation in the porous media [21,22], highlighting the adsorption on oxy-hydroxides of Fe, hematite nature and colloidal magnetite [23,24]. Also, the presence of carbonate complexes in high concentrations of U, when carbonate concentration is high, limits the adsorption of U. The U also has a high affinity for organic matter and can be sorbed by the soil humus, and other substances such as peat and coal, reaching in microorganisms high concentrations [6]. Besides hydrogeochemical parameters and properties derived from hostrock, the U content in groundwater may be influenced by the residence times, showing that dissolved U concentrations in an aquifer can grow linearly with the age of the water [25].

On the other hand, mercury poses perhaps the most significant hazard to human health and the environment due to its ability to bioaccumulate, their toxic effects and its possibility to mobilize in concentrations above most drinking water standards [26,27,28]. The main mercury-related human health concern is exposure to the highly neurotoxic organomercury species [29]. Mercury is scarce in the lithosphere, with an average elemental concentration of 0.08 mg/kg and 0.03 mg/kg in soils [30,31], although local variations may be significant. Inorganic mercury may show three states of oxidation: metallic Hg^0^, Hg^+^ (mercurous) and Hg^2+^ (mercuric). Metallic Hg^0^ and Hg^+^ may be oxidized to Hg^2+^, the most abundant species. Dissolved Hg takes several chemical forms [32]: elemental mercury (Hg^0^aq), which is volatile; a number of mercury species (Hg^2+^) that are complexed in variable amounts; monovalent Hg^+^; and organic mercury, such as methyl (MeHg), dimethyl (Me_2_Hg) and some forms of ethyl (EtHg) mercury.

The most soluble species is Hg^2+^, which complexes with Cl^−^ in oxidizing environments, thus enhancing its solubility [33]. Under aerobic conditions, the dominant inorganic mercury species are HgCl_2_ (at low pH), HgClOH (at neutral pH), and Hg(OH)_2_ (at high pH). Sediments showed a strong correlation between organic matter and the concentration of Hg. This indicates that soluble organic matter (SOM) may provide a large-surface-area substrate, which may act as a concentrator for Hg and other organic-associated elements [34]. In general, organic matter is an important factor in controlling mercury sorption in soils and sediments dominated by Hg-hydroxyl species [35]. The normal concentration of Hg in natural water is less than 1 μg/L and the lethal dose for humans is considered to be approximately 30 μg/day. Under normal groundwater conditions, the most stable state is the one that corresponds to Hg^0^ [36,37].

This study was focused on the geochemical processes that affect Uranium and Mercury concentrations in granitic and alluvial aquifers of Ridaura basin and the main objectives were:

- Evaluate the concentration and possible anomalies of Uranium and Mercury in rocks, mineralizations and sediments.
- Characterize the geochemical behavior of Uranium and Mercury along the groundwater flow in the Ridaura aquifers.
- Evaluate the possible controls of their geochemical behavior.

## 2. Materials and Methods

### 2.1. Study Area

**Figure 1:**
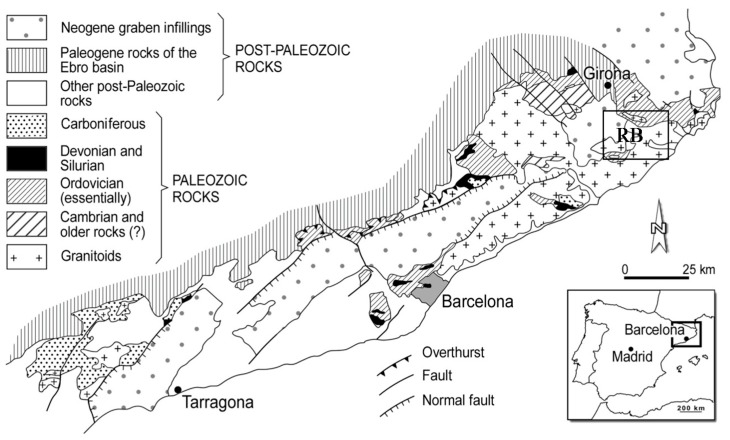
Geological map of Catalan Coastal Ranges, RB: Ridaura basin.

The study area is located in the NE of the Catalan Coastal Ranges (CCR), which consists of two mountainous alignments N60E direction and an elongated basin filled by Tertiary sediments, lava flows, basaltic dikes and necks and hydrothermal volcanic materials belonging to the Quaternary, and a set of alluvial terraces, which are the most important aquifers in the region [38,39,40,41] (Figure 1). The Paleozoic materials of the CCR are associated to metasedimentary rocks from Cambro-Ordovician to lower Carboniferous and a granite batholiths which outcrops over an area of more than 1500 km^2^ [42]. The granitic rocks in the studied area are, mainly, associated with biotite granodiorites, K-feldspar megacrysts-bearing biotite granodiorites, biotite granites and leucogranites. All granitic rocks are pre-Triassic, generally post-tectonic and younger than the regional metamorphism, and could be considered as late-Hercynian.

Geomorphologically, the studied area constitutes a “basin and range” structural area created during the distensive period following the Alpine orogeny, where the ranges consists of Paleozoic igneous and metamorphic rocks in the Montseny-Guilleries, Selva and Selva Marítima ranges. The basin areas consist of Pliocene sediments in the Selva basin and Quaternary alluvial deposits associated with the main rivers, especially the Ridaura River. Enclosed within the Paleozoic materials some mineralized veins are found in this area, which are characterized by a metal poor content and a fluorite dominant content. This region is characterized by a Mediterranean climate with an average temperature close to 14.5 °C, and an average annual rainfall of 772.7 mm which is concentrated in spring and fall.

**Figure 2:**
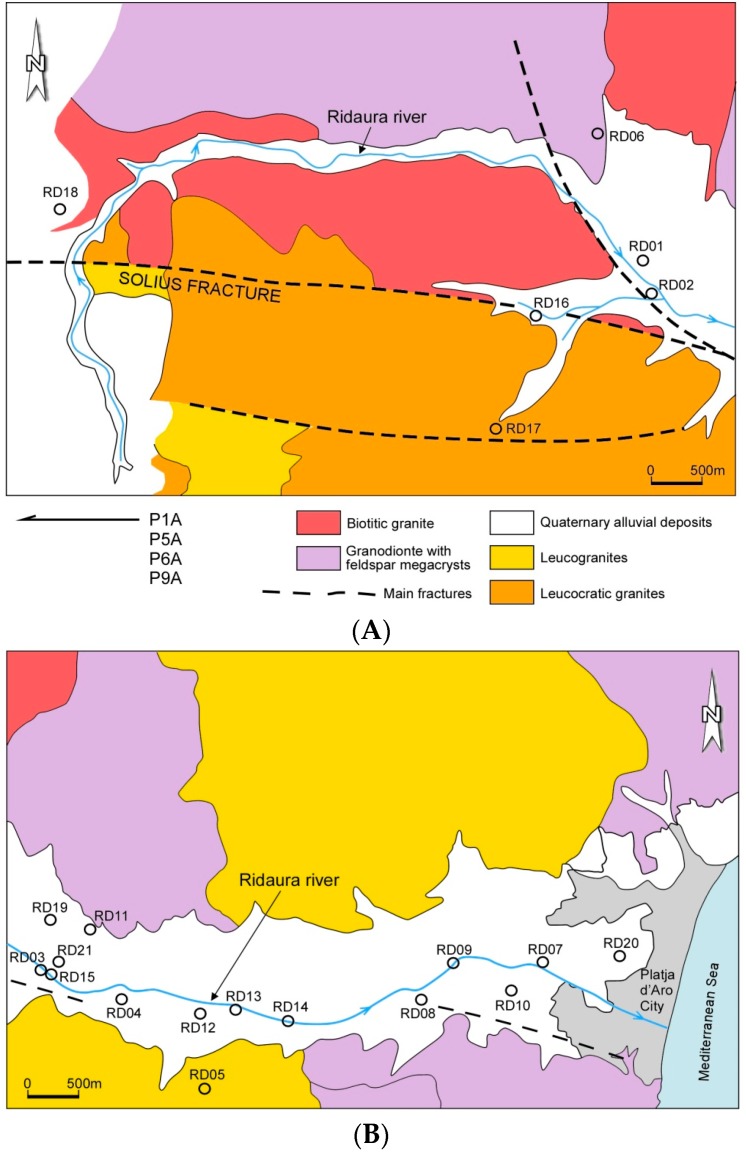
Synthetic geological map and water samples location. (**A**) Western area of Ridaura basin; (**B**) Eastern area of Ridaura basin.

In the study area, there are three main aquifers. The first unit comprises the deep regional aquifer contained within the fractured granitic basement. The second unit is associated with the weathered granitic materials and may recharge the deep aquifer and the alluvial system. The third is the system of shallow unconsolidated aquifers, which consist of recent colluvial/alluvial sediments associated with the Ridaura river (Figure 2A,B). The most permeable aquifers throughout this region are associated with Quaternary alluvial deposits. Granite materials, however, they are generally impermeable, allowing water circulation solely through superficially altered granodiorite and the fractured system that develops in these rocks. In the superficial deposits of altered granite, hydraulic conductivity can be high on the surface, decreasing rapidly with depth. The deep granite, slightly altered, is highly impermeable, circulating water solely by existing fractures, which gives rise to different storage systems and permeability. In the granitic system, recharging of the aquifer appears to occur in areas of high topography and discharge to the depressions and alluvial aquifers, giving rise to what is known as type flow system “basin and range”. Transit times of groundwater by metasedimentary rocks and granitic materials, from isotopic data, suggest transits over 50 years, for samples from La Selva and Gavarres [41]. Furthermore, the values in deuterium and O-18 showed, for water from deep wells in the granite, the possible recharge from areas of high topography and the possible existence of regional groundwater flow system.

The alluvial aquifer shows an area of 8 km^2^ located in the Ridaura basin (72 km^2^). The aquifer is wedged between granite reliefs of the northern end of the coastal range and is associated with Quaternary alluvial sediments and glacis and alluvial fans from weathered granite. These materials are sequences of sands, clays and, to a lesser extent, gravel, resulting from erosion and transport of granite substrate, forming a complex unit, with a thickness comprised between 15 and 25 m. Ridaura alluvial aquifer consists of two units (unconfined superficial aquifer and partially confined deep aquifer) hydraulically connected in some areas. These two aquifers are separated by an aqüitard of variable thickness.

The Ridaura alluvial aquifer is exploited to water supply at municipalities of Santa Cristina d’Aro, Castell-Platja d’Aro and Sant Feliu de Guixols (Figure 2B). The river Ridaura greatly affects the behavior of the groundwater flow, and can act as an element of discharge or recharge the aquifer, depending mainly of the piezometric level. The spatial distribution of hydraulic parameters is similar to that observed in previous studies, showing an area of high aquifer hydraulic conductivity in the sector close to the Ridaura River.

### 2.2. Sampling and Analysis

**Figure 3:**
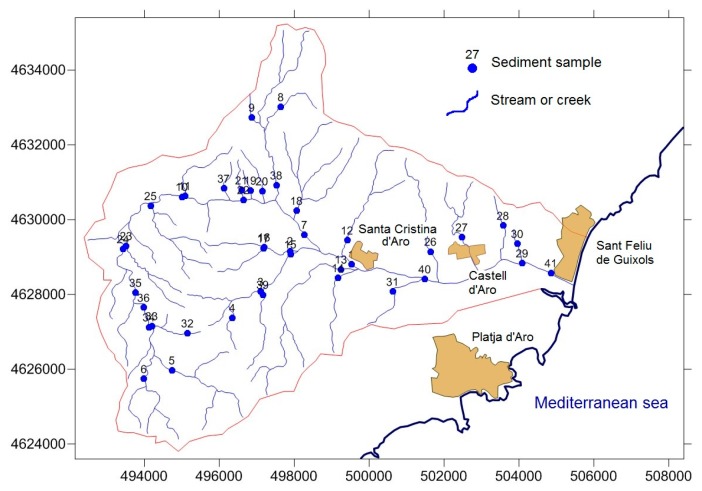
Location of sediment samples.

The mineralizations, sediments, and host rock were manually extracted to obtain approximately 1.5 kg of samples comprised of 15 samples from mineralizations and granitic rocks and 41 sediments (Figure 3). Solid samples were passed through a jaw crusher to a particle size of 10 meshes, quartered, pulverized in an agate mortar, rehomogenized, and repacked in plastic bags. Au, As, Ba, Br, Ce, Co, Cr, Cs, Eu, Fe, Hf, Hg, Ir, La, Lu, Na, Nd, Rb, Sb, Sc, Se, Sm, Sn, Sr, Ta, Th, Tb, U, W, Y, and Yb were quantitatively analyzed by instrumental neutron activation analysis (INAA), which involves bombarding the unaltered samples with neutrons. Mo, Cu, Pb, Zn, Ag, Ni, Mn, Sr, Cd, Bi, V, Ca, P, Mg, Tl, Al, K, Y, and Be were analyzed using inductively coupled plasma optical emission spectrometry (ICP-OES). These analyses used a process digestion, employing HF, HClO_4_, HNO_3_, and HCl to get as much of the sample into solution as possible; the resulting metals were determined by ICP-OES at Actlabs (Ancaster, Ontario, Canada).

Groundwater samples were collected from water-supply and agricultural wells located in the Ridaura alluvial aquifer (samples RD01 to RD21), from deep wells at the western border in the granite aquifer (samples P-1A, P-5A, P-6A and P-9A), in the Ridaura river (sample RD15) and two samples of rich-U mineral water from the nearby Montseny range was analyzed in order to compare with the groundwater sampled (samples OS12 and 13) (Figure 2A,B). The pH, redox potential (Eh, mV), temperature, and electrical conductivity (EC, μS/cm) were corrected using standard solutions and measured in-situ with portable devices (HACH sensION™378). The groundwater samples were filtered with a cellulose nitrate membrane with a pore size of 0.45 μm.

The samples for cation analysis were later acidified to pH < 2.0 by adding ultrapure HNO_3_. The samples were collected in 110-mL high-density polypropylene bottles, sealed with a double cap and stored in a refrigerator until analysis. The groundwater samples were obtained after purging each well using a bailer sampler and the submersible pumps of public supply and agricultural wells. The metal concentrations were measured using inductively coupled plasma-mass spectrometry (ICP-MS) at the Actlabs laboratories. The concentrations of chloride, nitrate, and sulfate (in a second untreated sample) were analyzed by ion chromatography. The alkalinity of some waters was analyzed by titration. The National Institute of Standards and Technology (NIST) standard reference material 1640 (ICP-MS) was used to confirm accuracy.

The hydrogeochemical analyses of groundwater were performed using GBW [43] and the PHREEQC numerical code [20] to evaluate the speciation of dissolved constituents and calculate the saturation state of the phase minerals. The Minteq thermodynamic database [44] was used for the chemical equilibrium calculations. Factor analysis (FA) has been used as a useful statistical method for detecting the structure in the relationships among hydrogeochemical variables [45,46]. FA was thus used to investigate the relationships among 25 variables of the sediments and groundwater (26 variables) in order to evaluate the origin of the metals.

## 3. Results

### 3.1. Geochemistry of Rocks, Mineralizations and Sediments

The results from analysis of metals and some major elements from six igneous rocks (GB, EPIS, K-F, APL, GMKC, APL and G), six intragranitic quartz-vein deposits (UVQ-1, UVQ-2, VQB-1–3, VQCC) and three polymetallic vein deposits (CMV-1 and CMV-2 samples associated to Fe-Ba deposits and CM associated to Pb vein deposits) located in the metamorphic aureole are presented in Table 1. On the basis of data of major elements the composition of igneous rocks generally fall within or near the normal range of granodiorites. Metals and trace elements reveals notable differences amongst both igneous rocks and vein deposits, although there are a clearly anomaly in Ni, which showed concentrations of 70–201 ppm in granodiorites, above the mean content of granites (0.5 ppm in Table 1) [47]. Also, vein deposits showed high Ni concentrations, mainly quartz veins and Th-U mineralized veins. The concentration of As, Sb and Cr is, also, anomalous in igneous rocks and quartz veins, above the mean content of granites (Table 1), especially the Cr content which reaches 89–155 ppm in granodiorites and 120–472 ppm in quartz-vein mineralizations.

The results of the analysis of mineralization and rocks (Table 1) showed only anomalous values in Th and U associated to quartz-vein deposits located into biotitic granite host-rock (12.6–42.4 and 2.2–23.3 ppm, respectively), while granodiorites showed low concentrations of these elements (3.1–5.9 ppm). Also, Th and U concentrations are low in the polymetallic vein mineralizations (samples CMV-1, CMV-2, CM) and aplitic dykes, where U reaches 2.8 ppm.

Trace-element concentrations in streambed sediments are influenced by the input of material eroded upstream of the sample site as well as by the formation and deposition of colloidal material during periods of low stream-flow, reflecting the geochemistry of the igneous rocks (Table 2). The mean concentration of Pb and Cr are situated near the concentration of U.S. Freshwater Sediment Screening Benchmarks (FSSB in Table 2) and mean Mn concentration is located above this limit. The content of the remainder elements is congruent with the normal geochemistry of granitic host-rock. Also, were used statistical methods to calculate background values of sediment chemical parameters [48]. The first method was the iterative 2-σ technique and the second the calculated distribution function method [48]. The results showed a geochemical background of 8.7–51.7 ppm for Hg and a geochemical background of 1.9–9.2 ppm for U. The sediment samples with anomalous contents in U were located in leucocratic and biotitic granites (samples 33, 35, 36 and 39) and granodiorites (samples 37 and 38) (Figure 3). However, the sediment samples with anomalous contents in Hg were related with leucocratic granite and granodiorite with the exception of sample 30 located in leucogranites.

### 3.2. Hydrogeochemistry

**Figure 4:**
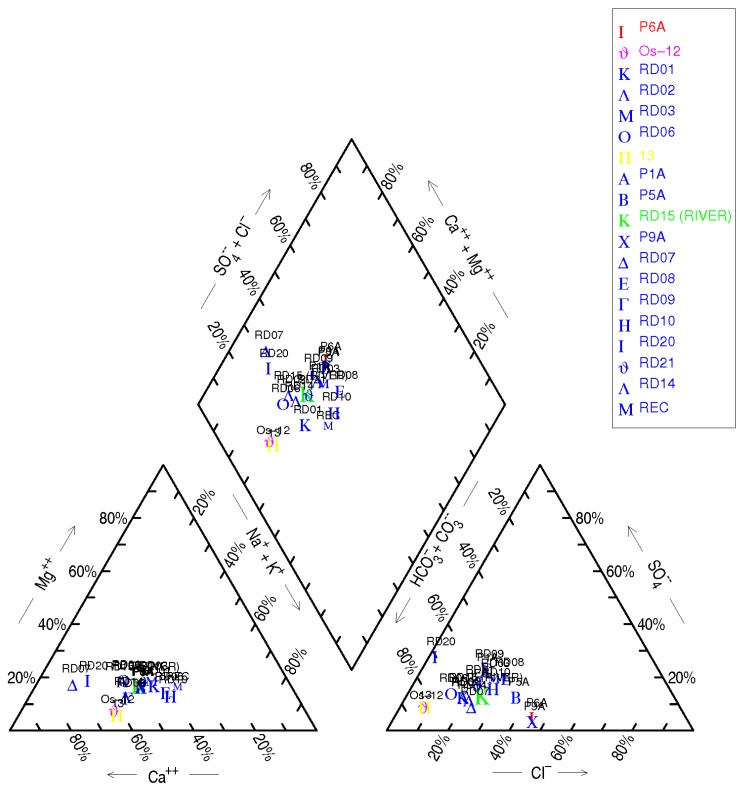
Piper diagram.

**Figure 5:**
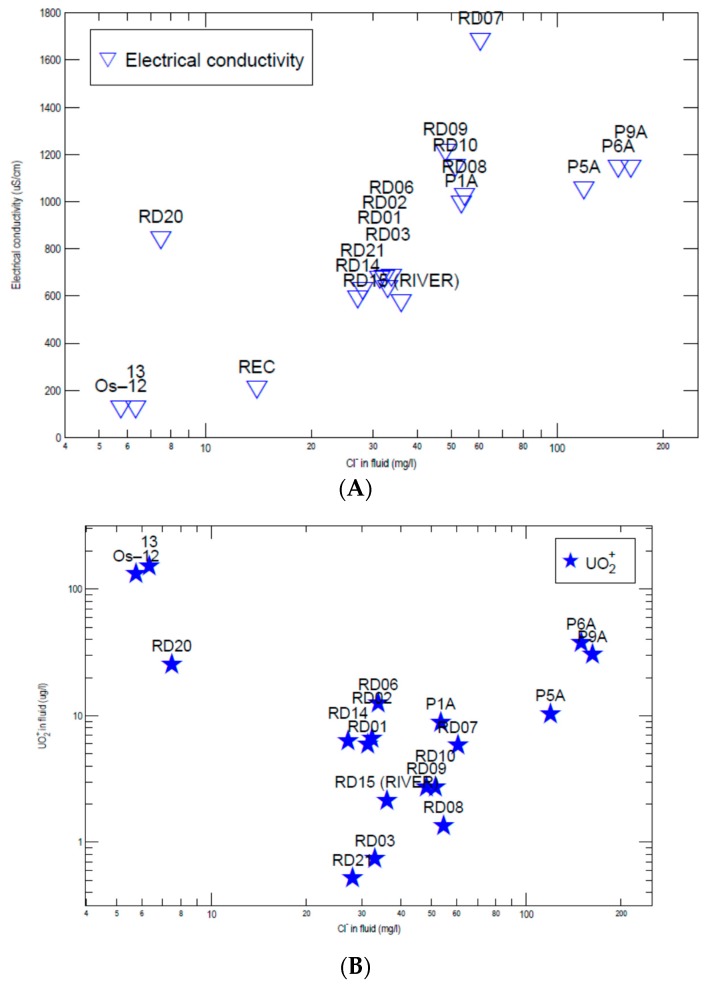
Evolution of electrical conductivity (**A**) and U (**B**) against chloride concentration.

Groundwater and surface water samples (Figure 2A,B) showed a relative hydrogeochemical homogeneity, with most of the samples belonging to the calcium bicarbonate facies (Table 3 and Table 4). Water types vary from Ca-Cl (P6A) and Na-HCO_3_ to Ca-HCO_3_ types, which represent the most of Ridaura samples. The electrical conductivity of groundwater showed levels comprised between 500 and 600 μS/cm increasing slightly until 600–680 μS/cm in the wastewater plant area located at Castell d’Aro town. Parallel to the right bank of the aquifer, higher conductivities were observed, with values comprised between 800 and 940 μS/cm. From Castell d’Aro area, there is a remarkable increase in electrical conductivity due to discharges from the wastewater treatment plant, and seawater intrusion in the central part of the aquifer (Platja D’Aro town).The spatial distribution of conductivity and the variation in the concentration of major elements, most likely indicates that there is an lateral entry of groundwater into the aquifer Ridaura from the granitic massif (left bank of Ridaura). In the Piper diagram (Figure 4) granitic deep waters (samples P1A, P5A, P6A and P9A), alluvial and superficial waters (RD15), uranium rich waters of nearly Montseny range (OS12 and 13) and recharge water in this area (REC sample) were represented. The Piper diagram showed the variability in their chemical composition, from deep waters to lower salinity waters represented by Montseny uranium-rich waters (OS12 and 13). In an intermediate location, the alluvial and superficial Ridaura samples were situated, which showed a similar chemical composition. The distribution of U in groundwater showed the most elevated contents in the deep wells exploiting the granitic aquifer (P1A, P5A, P6A and P9A), where uranium reaches 37.7 μg/L. Also, mineral water from the nearby Montseny range, which were used in human consumption, showed high concentrations comprised between 132 and 152 μg/L (Table 3), which are above the WHO [3] guidelines (30 μg/L). Thus, in Germany, bottled mineral water [16] showed detected U concentrations comprised between <0.0005 and 16.0 µg/L (median: 0.17 µg/L for 908 samples), while in Britain bottled waters [17] the highest concentrations of U are associated with sandstone aquifers of Permotrias and Devonian age. In contrast, bottled water from Norway, Sweden, Finland and Iceland showed high concentrations of U (29–32.4 µg/L) from wells located in granite and similar materials [18]. In the alluvial Ridaura aquifer, the concentration of U was comprised between 0.7 and 25.5 μg/L (Table 3), showing significant amounts in the samples RD06, RD01 and RD02, located close to a fracture in the direction NW-SE. Geochemical background of U in groundwater was comprised between 0.2 and 11.3 μg/L and the samples above the threshold limit were deep granite waters (samples P6A and P9A), RD06 and RD20. Electrical conductivity reflects the low salinity of most of the samples (Table 4) and Figure 5A showed the increase produced in electrical conductivity based on the chloride content from the recharge water and leading to the richer salinity samples as are the deep granite samples. Besides, there is a possible relationship between salinity and U concentration, showed in Figure 5B. Increased mineralization and U concentrations could be produced by water-rock interaction during groundwater flow.

The distribution of Hg showed concentrations above the European guidelines (1 μg/L) in 10 alluvial samples (Table 3). Geochemical background of Hg in groundwater was comprised between 0.2 and 1.8 μg/L and the samples above the threshold limit were RD07, RD09, RD14 and RD19. The greater concentration was associated to RD14 and RD19 samples (Figure 3), located near the limit of alluvial aquifer with igneous rocks, which suggest a possible migration of Hg from granitic materials. Also, some samples located in the deltaic area (samples RD07 and RD09) showed high concentrations of Hg, in a region of intense agricultural activity.

Thus, some samples, and the samples with greater Hg content (RD11, RD14 and RD19), are located near golf courses and residential areas, where mercury is frequently applied as fungicide [27]. Mercurial compounds were used on golf facilities, estimating an annual application in USA of 2.1 kg of Hg per hectare [28]. Thus, mobilization of Hg from applications of mercurial compounds, enhanced by subsequent disturbance from residential developments, may cause elevated Hg concentrations on groundwater [27]. The migration of Hg could be originated by leaching of Hg from these areas located over weathered granite and the possible flux of contaminated runoff to the alluvial deposits. Besides, the hydraulic connection between superficial weathered granite and alluvial aquifer may explain a continuous flux of Hg-rich groundwater to alluvial system, at least in the geochemical anomalous areas.

On the other hand, RD16 and RD18 samples and the samples located in the deltaic area (RD07 and RD09) are associated with agricultural zones. Thus, Barringer et al. [28] suggested that Hg could be introduced in soils and groundwater via fertilizers, since commercial fertilizer solution may contain 280 μg/L of Hg. Besides, Barringer et al. [27] indicated that the highest Hg concentration (5.1 ppm) in fertilizers may be associated with calcium superphosphate and a lower concentration (1.2 ppm) in Nitrogen-Phosphorus-Potassium (NPK) fertilizer. Also, in the New Jersey Coastal Palin aquifer system, shallow groundwater with high contents of chloride, nitrate and Hg (5 μg/L) may be polluted by the use of fertilizers or from septic tanks [49]. In this sense, samples RD07, RD09, RD16 and RD18 showed relatively high contents of chloride and nitrate, which may indicate inputs from septic-systems effluent or fertilizer applications.

### 3.3. Geochemical Modeling

The PHREEQC code [20] and code GWB [43] were used to determine the dominant hydrochemical species in waters with anomalous uranium values and for studying the speciation of uranium in groundwater, according to the conditions of pH and Eh.

Regarding the dominant species, data from samples with a higher concentration of uranium indicate that the dominant uranium species are UO_2_(CO_3_)_2_^2−^ and UO_2_(CO_3_)_3_^4−^. Besides, groundwater was undersaturated with respect to some mineral phases as petchblende, autunite, and various oxides of U.

**Figure 6:**
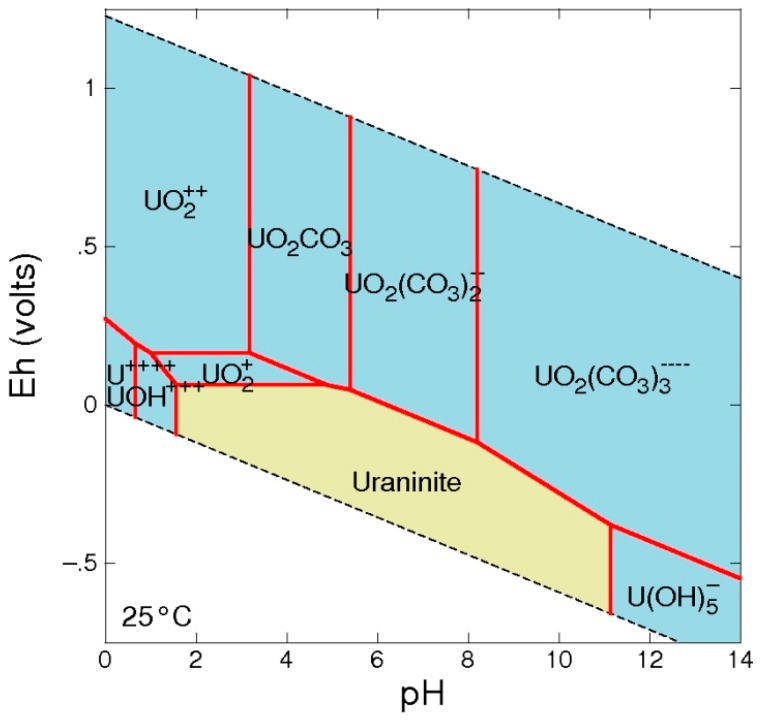
Eh-pH diagram of uranium. Diagram U^++++^, *T* = 25 °C, *P* = 1 bar, a [main] = 10^−10^, a [H_2_O] = 1, a [HCO_3_^−^] = 10^−2^.

On the other hand, according to the pH conditions of the aquifer, the GWB code confirms that the more stable species is carbonate complex UO_2_(CO_3_)_2_^2−^ (Figure 6), although under alkaline pH, the dominant species becomes UO_2_(CO_3_)_3_^4−^ All this would indicate the mobility of uranium (VI) in water and its oxidized neutral-alkaline character similar to most of the sampled waters.

### 3.4. Multivariate Analysis

**Figure 7:**
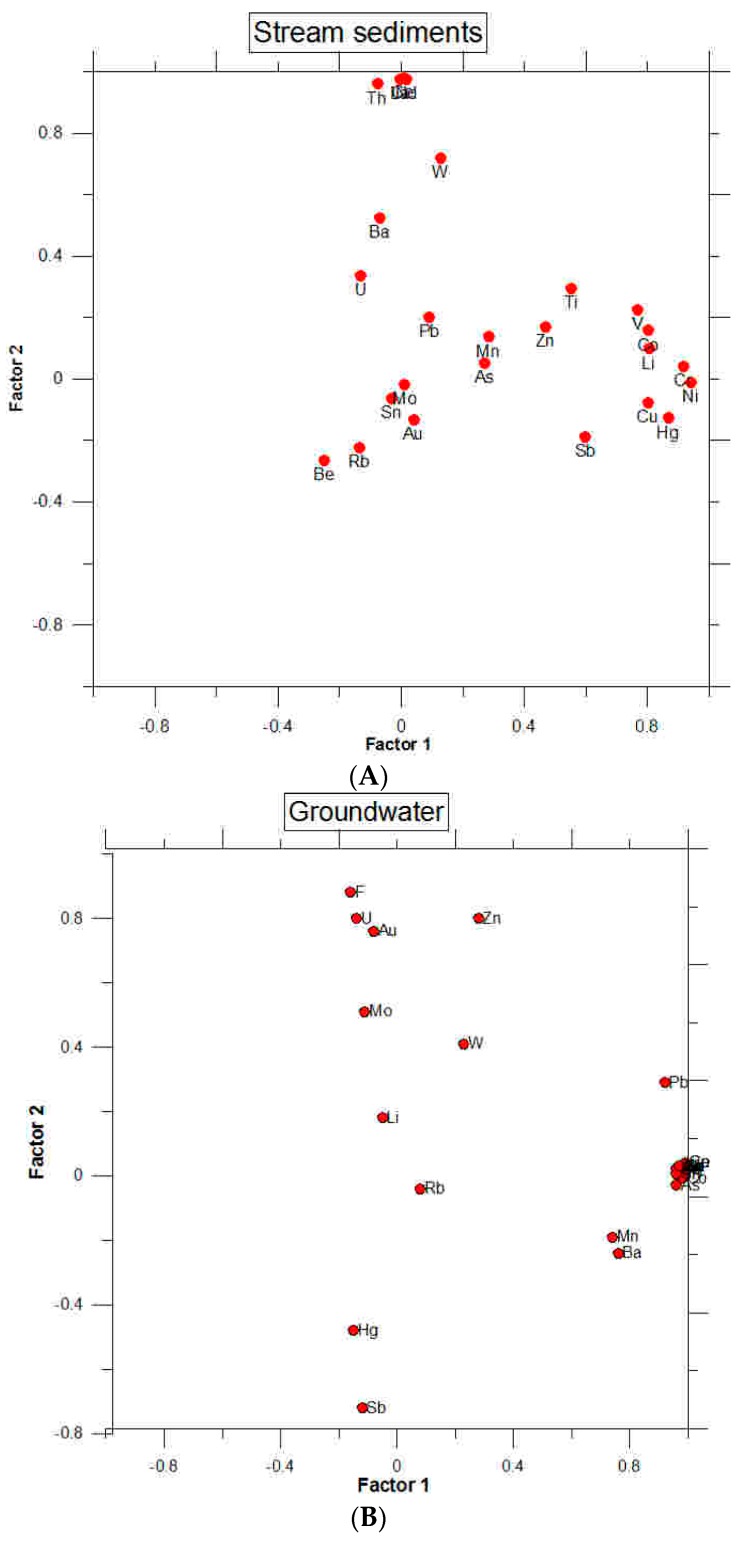
Factorial diagram for sediments (**A**) and groundwater (**B**).

Factorial analysis (varimax normalized method) of sediments and 25 variables (Hg, Au, Cu, Mo, Pb, Ni, Zn, As, Ba, Be, Co, Cr, Li, Mn, Rb, Sb, Ti, Th, U, V, W, La, Ce, Nd and Sn) showed four factors that explained about 78% of the total variance (Table 5). Factor I explains 24.7% of the total variance, and appears to represent a possible “mineralization” factor, since it is strongly correlated with the elements: Hg, Cu, Ni, Co, Cr, Li and V, and possibly with Sb and Ti (Figure 7).

The second factor is associated with Th, W, La, Ce and Nd and accounts for about 13.9% of the total variance. Factor II appears to be related to REE and W, which is consistent with the possible presence of monazite rich Th in the granitic rocks.

The third factor is associated with U, Be and Rb, which also are anomalous in rich-U mineralizations and host-rock (Table 1). This factor may explain the origin of uranium anomalies in quartz mineralizations, where these elements showed high concentration. The association U-Be-Rb may be also associated to the presence of pegmatites, relatively frequent in this area. The fourth factor is mainly composed of Mo and As, which may be associated with possible high temperature mineralizations of molibdenite and arsenopyrite. Since mineralizations of granitic rocks showed higher Th/U ratios (>2), the uranium presence may correspond to uranium mostly localized in refractory sites (monazite, zircon, apatite, etc.) [50].

Factorial analysis of groundwater using data from 25 water samples (alluvial and granitic samples) and 26 variables showed the association of metals with possible geochemical processes. The variables included Hg, Au, Cu, Mo, Pb, Ni, Zn, As, Ba, Co, Cr, Li, Mn, Rb, Sb, Ti, Th, U, W, La, Ce Nd and F. The results (Table 6) showed four independent factors, which account for 84% of the total variance.

The first factor is responsible for 56.8% of the total variance and is best represented by Be, Ti, V, Cr, Mn, Co, Ni, Cu, As, Sn, Ba, La, Ce, Nd, Pb and Th. This factor seems to be related to weathering of host-rock and mineralizations, since it is associated with a majority of elements, constituting a “mineralization” factor of groundwater (Figure 7).

Factor 2 explains 15.7% of the total variance and it is represented by Zn, U, F and inversely with Hg and Sb. This factor could be related to uranium mobilization in groundwater, which also seems related to F and Zn mobilization. In fact, in the Montseny-Guilleries area, the uranium anomalies in groundwater are related with high contents of Zn.

Factor 3 is responsible for 6.3% of the total variance and shows Rb and Mo to be negatively correlated. Factor 4 explains 6.3% of the total variance and is represented by W and Li and could be a mineralization factor associated with the weathering of high-temperature granitic mineralizations.

## 4. Conclusions

Results from this study indicated that U content of major lithologies, mineralizations and sediments reveals that none are extremely enriched uranium. Also, results implicate certain lithologies (biotitic granites, leucocratic granites) as likely sources of uranium in groundwater. In relation to the uranium contents detected in groundwater, the highest concentrations occur in some samples of mineral water of the nearby Montseny massif, which exceed the 132 μg/L and supply wells of more than 100 m deep located in the granitic aquifer of the Ridaura aquifer west border, where concentrations have reached 37.7 μg/L. Granitic rocks contain minerals that, possibly, have uranium as a minor constituent and weathering of these minerals may release uranium to water.

The relationship between salinity and U contents suggests that deep groundwater flowing into the granitic system may contain high uranium concentrations and could inflow into alluvial aquifer by fractures, increasing the dissolved uranium of alluvial groundwater. Multivariate analysis of sediment samples showed a factor associated with U, Be and Rb, which may explain the origin of uranium anomalies in quartz mineralizations located in biotitic granites and leucocratic granites, where these elements showed a significant concentration. The association U-Be-Rb may be also associated with the presence of pegmatites, relatively frequent in this area. Multivariate analysis of groundwater showed a factor represented by Zn, U, F and inversely with Hg and Sb. This factor could be related to uranium mobilization in groundwater, which also seems to be related to F and Zn mobilization. Thus, based on the data gathered in this study, the occurrence of U in groundwater appears to be controlled, predominantly, by anomalous presence of U in the different major rock types of the region and the regional and/or local hydrogeological setting of the various parts of the alluvial and granitic aquifer.

Spatial distribution of U in groundwater and geochemical evolution of groundwater suggest that U levels appear to be highest in granitic areas where groundwater has long residence times and a significant salinity. The presence of high U concentrations in alluvial groundwater samples could be associated with hydraulic connection through fractures between alluvial system and deep granite system (samples RD01, RD02 and RD06). According to this model, oxidizing groundwater moving through fractures in the leucocratic/biotitic granite containing anomalous U contents are the most likely to acquire high levels of U.

The distribution of Hg in groundwater showed concentrations above the European guidelines (1 μg/L) in 10 alluvial samples. The greater concentration was associated with samples located near the limit of the alluvial aquifer with igneous rocks, which suggests a possible migration of Hg from granitic materials. Also, these samples are located near golf courses and residential areas, where mercury is frequently applied as fungicide. The presence of high concentrations of Hg in groundwater samples of agricultural areas could be associated with the addition of fertilizers. In this sense, groundwater samples with relatively high contents of Hg, chloride and nitrate may indicate inputs from septic-systems’ effluent or fertilizer applications.

Therefore, the compiled data indicated that drinking water from crystalline bedrock and alluvial aquifers should be analyzed in detail. In case of elevated concentrations of U and/or Hg, treatment alternatives should be considered.

## Figures and Tables

**Table 1:** Geochemistry of igneous rocks and mineralizations.

Sample	Au	Ag	Cu	Cd	Mo	Pb	Ni	Zn	As	Ba	Be	Bi	Ca	Co	Cr	Eu	Fe	Hg	Mn	Rb	Sb	Se	Ta	Th	U
Unit	ppb	ppm	ppm	ppm	ppm	ppm	ppm	ppm	ppm	ppm	ppm	ppm	%	ppm	ppm	ppm	%	ppm	ppm	ppm	ppm	ppm	ppm	ppm	ppm
DL	2	0.3	1	0.3	1	3	1	1	0.5	50	1	2	0.01	1	2	0.2	0.01	1	1	15	0.1	3	0.5	0.2	0.5
GB	<2	<0.3	57	<0.3	<1	7	201	90	<0.5	280	3	<2	3.07	11	89	1.2	3.79	<1	508	112	2.1	<3	<0.5	6.5	<0.5
UVQ-1	<2	<0.3	16	0.3	29	30	228	155	10.3	210	7	<2	0.05	4	120	0.4	7.86	<1	3860	230	2.3	<3	2.3	40.4	23.3
UVQ-2	<2	<0.3	13	0.5	19	33	581	125	7	280	5	<2	0.06	3	226	0.2	5.48	<1	2300	244	2.9	<3	2.9	42.4	17.1
EPIS	<2	<0.3	3	<0.3	1	12	248	70	2.4	<50	4	<2	3.55	7	97	1.2	2.41	<1	436	151	1.4	<3	<0.5	12.6	2.2
VQB-1	<2	<0.3	10	<0.3	4	<3	790	17	3.4	<50	<1	<2	0.02	1	333	<0.2	0.47	<1	39	<15	6	<3	<0.5	1.7	0.8
VQB-2	<2	<0.3	4	0.4	6	<3	841	10	2.3	<50	<1	<2	0.02	1	411	<0.2	0.26	<1	28	<15	3.9	<3	<0.5	0.5	<0.5
VQB-3	<2	<0.3	5	<0.3	5	5	705	8	0.8	<50	<1	<2	0.01	2	369	<0.2	0.35	<1	64	<15	0.1	<3	2.2	12.6	2.5
K-F	2	3.5	262	1.4	7	2270	63	113	59.2	1360	2	<2	2.3	3	192	1.4	1.59	<1	324	144	10.4	<3	<0.05	7.7	1.6
VQCC	<2	1.1	208	0.4	7	632	127	98	24.8	390	2	29	0.52	14	472	0.2	4.66	<1	736	136	5.5	<3	<0.05	2.9	1.0
GMKC	<2	<0.3	5	<0.3	<1	41	9	84	5.7	770	4	<2	1.5	11	19	0.9	2.24	<1	3500	182	1.1	<3	<0.5	13.7	3.1
APL	<2	<0.3	6	<0.3	1	8	3	22	6.1	580	2	<2	0.31	3	<2	0.7	0.98	<1	1600	98	1.1	<3	1.5	8.0	2.8
G	<2	<0.3	8	0.5	<1	38	70	116	4.8	710	3	<2	1.32	19	155	2.3	5.03	<1	1050	121	2.1	<3	3.4	14.7	5.9
CMV-1	3	<0.3	134	1.3	41	77	131	97	5.8	3100	2	<2	1.3	40	110	0.9	6.88	<1	236	250	4.3	<3	1.1	11.3	6.0
CMV-2	<2	0.5	17	0.8	<1	5	78	72	19.6	1850	2	4	26.8	20	42	0.8	2.13	<1	795	81	4.3	<3	<0.5	5.8	0.8
CM	14	1	60	0.7	57	22276	29	61	32.6	185	<1	<2	0.29	2	96	0.8	0.96	<1	145	19	4.0	<3	<0.5	0.7	6.8
AGR	4	0.04	10	0.2	2	20	0.5	40–100 *	1.5	600	5	0.1	---	1	4	1–2 *	1.4–2.7 *	0.08	500	150	0.2	0.05	3.5	17	2.5–6 *

DL: detection limit, GB: biotitic granodiorite, UVQ-1: granitic mineralized quartz vein, UVQ-2: granitic mineralized quartz vein, EPIS: episienites, VQB-1: Sant Baldiri quartz vein, VQB-2: Sant Baldiri quartz vein, VQB-3: Sant Baldiri quartz vein, K-F: K-feldspar megacryst, VQCC: mineralized quartz vein close to Can Carbonell town, GMKC: granodiorite with K-feldspar megacryst, APL: aplite dyke, G: granodiorite, CMV-1: Can Carbonell vein deposits, CMV-2: Can Carbonell vein deposits, CM: Can Magre vein deposits (F-Pb), AGR: abundance in granitic rocks [47], *: [6].

**Table 2:** Geochemistry of sediments.

Sample	Hg	Au	Cu	Mo	Pb	Ni	Zn	As	Ba	Be	Co	Cr	Li	Mn	Rb	Sb	Ti (%)	Th	U	V	W	La	Ce	Nd	Sn (%)
1	48	39	23	5	23	11	65	2.10	500	3	7	41	30	581	153	0.40	0.25	22.50	3.10	38	1.00	50	99	25	0.01
2	103	1	41	2	30	46	115	7.00	530	3	18	180	64	1000	162	1.20	0.41	27.90	3.50	91	15.00	67	135	37	0.01
3	37	1	22	4	26	13	46	4.90	390	4	5	41	41	473	297	0.60	0.53	29.70	7.60	63	1.00	35	80	22	0.01
4	47	1	6	1	38	5	41	3.60	350	6	4	20	23	421	360	0.60	0.10	36.90	8.70	15	1.00	38	90	33	0.01
5	23	1	4	1	23	4	25	2.40	50	6	4	16	21	349	288	0.40	0.10	22.50	6.00	12	1.00	17	43	7	0.01
6	23	1	4	2	32	3	31	0.50	330	6	1	1	21	612	279	0.10	0.06	18.90	5.00	7	1.00	14	42	5	0.01
7	41	1	12	6	33	11	85	4.80	50	3	9	39	37	953	153	0.70	0.38	45.00	6.40	52	32.00	108	198	74	0.01
8	37	8	15	6	34	9	79	3.50	630	5	9	30	45	640	90	0.50	0.29	39.60	3.30	46	1.00	117	207	68	0.01
9	34	1	11	4	30	24	84	3.20	640	4	11	36	48	840	180	0.60	0.45	28.80	4.10	64	1.00	68	135	46	0.01
10	26	1	11	3	28	9	71	4.60	530	4	5	24	34	570	162	0.40	0.26	33.30	3.90	42	1.00	70	135	38	0.01
11	14	1	13	1	27	7	116	2.70	610	3	5	23	31	597	189	0.10	0.35	82.80	7.80	41	1.00	171	306	90	0.01
12	28	5	7	1	26	5	48	2.00	50	3	5	1	28	674	144	0.40	0.22	28.80	3.80	28	1.00	55	117	33	0.01
13	24	9	9	1	27	8	32	4.10	320	4	3	31	23	314	225	0.60	0.14	20.70	5.80	21	6.00	26	59	14	0.01
14	45	1	12	2	28	10	49	4.70	280	4	6	24	29	402	189	0.60	0.16	31.50	8.40	25	1.00	34	88	28	0.01
15	33	1	12	1	30	11	44	4.10	270	5	5	24	37	292	234	0.50	0.16	32.40	8.30	27	1.00	37	90	32	0.01
16	103	6	119	1	34	45	114	2.90	50	3	16	180	74	1110	171	1.00	0.43	23.40	3.20	93	1.00	51	108	34	0.01
17	84	15	14	3	57	18	64	4.40	320	4	8	69	56	425	261	0.50	0.27	22.50	6.00	52	9.00	37	82	35	0.01
18	39	1	12	2	27	13	62	4.60	450	3	8	24	40	2070	108	0.50	0.32	33.30	4.40	46	9.00	71	135	41	0.01
19	47	1	20	7	33	12	90	0.50	560	4	11	28	55	981	153	0.80	0.45	31.50	5.70	65	1.00	77	153	43	0.01
20	19	1	14	1	29	7	59	2.40	420	3	5	14	35	551	171	0.50	0.13	31.50	4.10	19	1.00	63	117	42	0.01
21	19	1	25	1	35	11	92	2.30	610	3	7	19	43	1050	162	0.40	0.16	48.60	6.40	32	1.00	99	198	59	0.01
22	15	1	12	1	102	11	70	4.30	460	3	8	55	36	867	180	0.40	0.23	73.80	6.40	39	1.00	171	315	99	0.01
23	18	5	32	15	36	19	150	13.5	540	5	12	44	54	812	180	0.10	0.44	29.70	2.90	76	16.00	89	171	50	0.01
24	18	5	12	3	36	10	74	4.80	480	5	6	18	33	634	198	0.40	0.23	19.80	4.30	39	11.00	36	73	22	0.01
25	28	14	15	9	37	14	103	6.30	430	4	9	37	49	795	171	0.60	0.28	35.10	6.40	51	1.00	61	126	44	0.01
26	19	1	14	1	30	10	64	2.70	410	4	4	15	38	416	234	1.40	0.20	16.20	6.50	25	1.00	30	64	16	0.01
27	46	1	12	1	35	7	76	2.60	360	4	3	11	50	583	252	0.70	0.16	11.70	5.70	21	1.00	23	47	18	0.01
28	17	1	9	8	22	12	66	0.50	350	3	6	23	41	526	198	0.60	0.33	15.30	3.40	56	1.00	32	68	23	0.01
29	28	1	11	2	26	7	56	0.50	650	3	5	35	29	571	180	0.60	0.27	30.60	5.40	36	10.00	73	144	41	0.01
30	63	1	82	1	40	16	76	6.00	430	4	6	34	46	470	225	1.00	0.31	12.60	3.60	54	1.00	33	67	14	0.01
31	26	1	13	1	30	11	63	4.70	400	4	5	20	41	367	171	0.90	0.16	27.00	6.80	23	19.00	38	90	29	0.01
32	35	1	10	2	30	15	48	6.80	50	5	7	27	33	402	207	0.90	0.25	24.30	7.70	42	10.00	35	85	30	0.01
33	36	64	25	1	59	19	157	7.80	470	5	6	51	40	810	230	1.20	0.23	45.00	10.00	35	8.00	41	94	30	0.01
34	13	1	2	1	23	4	23	2.60	50	6	3	1	20	348	310	0.10	0.07	25.00	8.70	10	1.00	13	38	11	0.01
35	56	1	15	2	31	14	87	4.10	460	6	6	38	41	1270	260	0.60	0.23	48.00	12.00	35	10.00	49	120	30	0.11
36	41	54	15	2	48	15	96	7.00	430	6	7	50	35	1120	240	0.80	0.21	44.00	10.00	36	1.00	38	93	24	0.01
37	58	1	29	6	31	12	84	5.50	500	4	9	20	51	868	150	0.60	0.19	52.00	14.00	41	1.00	100	180	66	0.01
38	24	1	13	1	32	11	70	3.50	880	3	7	33	41	628	190	0.40	0.39	170.00	11.00	58	51.00	390	700	200	0.01
39	38	1	9	1	38	10	47	3.10	350	7	5	23	38	958	250	0.10	0.16	59.00	16.00	22	1.00	55	110	45	0.01
40	27	1	11	1	30	9	64	2.20	410	3	5	24	31	701	160	0.30	0.22	24.00	3.10	37	1.00	42	84	22	0.09
41	43	1	25	4	27	21	118	5.20	450	4	10	37	53	572	220	0.80	0.39	27.00	3.10	61	1.00	48	100	34	0.01
M	37.1	6.1	18.8	2.8	33.9	12.9	73.2	4.0	402	4	6.8	35.6	39	698	203	0.58	0.25	36.1	6.4	40	5.7	65	131	40	0.01
FSSB	180	---	31.6	---	35.8	22.7	121	9.8	---	---	50	43.4	---	460	---	2	---	---	---	---	---	---	---	---	---

Values in ppm, Hg and Au in ppb. M: mean values. FSSB: U.S. EPA Freshwater Sediment Screening Benchmarks.

**Table 3:** Main anion contents of water samples.

Symbol	pH	Eh	EC	Cl	Br	NO_3_	SO_4_
Unit	pH Unit	mV	μS/cm	mg/L	mg/L	mg/L	mg/L
RD01	7.02	180	680	31.3	0.06	11.0	29.1
RD02	6.97	128	684	32.3	0.08	17.9	30.9
RD03	6.85	123	641	33	0.08	14.7	36.5
RD04	6.87	96	671	30.1	0.07	4.9	35.1
RD05	6.47	ND	464	27.8	0.06	16.6	29.4
RD06	6.66	378	690	33.8	0.07	12.7	47.5
RD07	6.72	156	1691	60.5	0.09	5.3	33.4
RD08	6.15	125	1033	54.5	0.14	4.7	50.9
RD09	6.62	100	1220	48.1	0.14	12.7	73.2
RD10	6.4	24	1154	51.3	0.12	1.3	42.1
RD11	ND	ND	ND	36.2	0.08	17.9	58.9
RD12	6.18	116	547	31.5	0.07	4.6	71.8
RD13	6.14	120	581	32.1	0.08	8.4	51.2
RD14	5.74	144	598	27.1	0.08	0.04	20.9
RD15	8.18	40	581	36	0.07	10.2	27.4
RD16	6.71	274	1093	41.5	0.07	5.8	48.9
RD17	7.7	235	140	19.5	0.05	8.0	14
RD18	7.85	263	833	18.6	0.07	7.1	19.8
RD19	7.13	247	912	3.81	<0.03	1.2	40.8
RD20	6.95	-105	850	7.47	0.05	3.6	94.8
RD21	6.76	17.8	632	28	0.07	6.0	31.9
P-1A	7.7	100	1000	53.4	0.2	0.08	76.8
P-5A	7.06	116	1060	119	0.41	13.4	58.2
P-6A	7.5	101	1020	149	0.65	0.6	24.3
P-9A	7.42	18	1150	162	0.71	0.1	19.4
OS-13	7.7	200	187	6.33	<0.03	0.1	10.2
OS-12	7.6	200	177	5.75	<0.03	0.1	9.87
EMLD	---	---	2500	250	---	50	250

EMLD: European primary drinking water regulations [4]. Values in mg/L. ND: non determined. RD: alluvial water samples, P-1A to P-9A: water samples from the Ridaura border belonging to granitic aquifer. OS-12 and OS-13: Mineral water from the nearby Montseny area.

**Table 4:** T Main metal contents of water samples.

Sample	Li	Be	Ti	V	Cr	Mn	Co	Ni	Cu	Zn	As	Rb	Mo	Sn	Sb	Ba	La	Ce	Nd	W	Au	Hg	Pb	Th	U	F
RD01	61	0.1	4.9	0.3	0.5	1.2	0.005	2.9	3.8	38	0.28	0.276	1.6	0.3	1.33	42.2	0.033	0.014	0.028	0.02	0.002	0.2	1.17	0.001	5.92	0.18
RD02	20	0.1	3	0.2	0.5	0.8	0.005	4.3	0.8	27.7	0.22	0.131	2	0.1	0.96	34.7	0.007	0.007	0.001	0.02	0.002	0.7	0.46	0.001	6.57	0.43
RD03	10	0.1	4.4	0.2	0.5	9.9	0.005	0.7	2.7	35.2	0.3	0.268	0.5	0.1	1.33	31.2	0.027	0.032	0.024	0.02	0.002	0.3	0.55	0.002	0.737	0.16
RD04	9	0.1	2.2	0.3	0.5	2.2	0.005	0.6	1.5	9.3	0.17	0.28	1.1	0.1	1.39	32.4	0.018	0.023	0.013	0.02	0.002	0.2	0.95	0.001	1.13	0.13
RD05	31	0.3	4.4	0.1	0.5	1.5	0.005	0.3	0.8	17.6	0.09	4.1	0.1	0.1	1.19	5.8	1.23	0.02	1.87	0.03	0.002	1.4	0.3	0.002	2.45	0.2
RD06	11	0.1	2.3	0.4	0.7	1.5	0.005	0.5	0.8	6.7	0.23	0.118	2	0.1	1.34	51.7	0.038	0.016	0.028	0.05	0.002	1.5	1.55	0.001	12.6	0.18
RD07	15	0.1	3.6	0.2	0.9	434	1.47	4.6	2.1	21.1	0.58	0.974	1.1	0.1	1.2	81.8	0.013	0.02	0.002	0.02	0.002	1.8	0.86	0.001	5.8	0.05
RD08	15	0.1	4.9	0.5	0.7	1260	0.671	5.1	6.1	33.8	1.11	1.02	3.3	0.2	1.42	120	0.073	0.12	0.058	0.05	0.002	0.5	1.54	0.005	1.35	0.09
RD09	19	0.1	5	1.1	0.8	620	0.086	2.1	1.8	28.1	0.71	1.17	1.5	0.1	1.43	106	0.027	0.029	0.01	0.03	0.002	1.8	0.7	0.001	2.73	0.08
RD10	26	0.9	66.1	16.3	8.8	1640	8.62	46	132	158	3.75	9.86	1.2	0.9	1.03	195	9.06	20.7	10.5	0.06	0.002	0.2	32.7	1.15	2.73	0.11
RD11	26	0.1	3.2	0.6	0.8	0.7	0.005	0.3	0.5	6.7	0.19	0.158	0.4	0.1	1.23	28.1	0.119	0.117	0.054	0.03	0.002	4.2	0.28	0.002	11	0.26
RD12	12	0.1	1.9	0.8	0.5	15.8	0.059	1	1.2	6.6	0.46	0.354	1.4	0.1	1.33	51.2	0.03	0.05	0.02	0.02	0.002	1.2	0.38	0.005	2.34	0.15
RD13	12	0.1	1	0.4	0.5	95.6	0.799	1	1.1	7.2	0.54	0.819	2.3	0.1	1.26	46.2	0.017	0.023	0.001	0.02	0.002	0.3	0.3	0.001	2.18	0.15
RD14	31	0.1	2	0.1	0.5	823	0.224	2.4	0.8	4.6	0.51	3.46	4.7	0.1	1.89	30.3	0.004	0.008	0.001	0.03	0.002	3	0.25	0.001	6.35	0.23
RD15	12	0.1	0.2	0.2	0.5	4.5	0.005	0.3	0.7	4.6	0.18	0.373	1	0.1	1.28	37.8	0.022	0.013	0.01	0.02	0.002	1.1	0.23	0.001	2.13	0.15
RD16	16	0.1	1	0.4	0.5	5.9	0.005	1.7	1.1	30.2	0.45	0.896	0.7	0.1	1.28	69	0.017	0.004	0.001	0.02	0.002	1.7	0.04	0.001	5.53	0.07
RD17	3	0.1	7.2	0.4	0.6	3.7	0.05	0.8	2	8.4	0.22	1.43	0.4	0.1	1.39	6.4	0.13	0.425	0.163	0.02	0.002	0.3	0.99	0.245	0.258	0.34
RD18	155	0.1	1.1	0.3	0.5	0.1	0.005	0.4	0.8	26.3	0.13	10.7	3.7	0.1	1.08	52.3	0.004	0.005	0.001	0.09	0.002	0.9	0.05	0.001	9.06	0.1
RD19	13	0.1	0.5	0.1	0.5	2.1	0.005	1.4	1.7	8.7	0.13	66.9	7.1	0.1	1.35	90.8	0.01	0.016	0.001	0.02	0.002	2	0.39	0.001	7.29	0.08
RD20	6	0.1	0.1	0.2	2.2	510	0.005	1.5	1.3	16.8	0.62	0.252	1.6	0.1	0.87	33.3	0.005	0.002	0.001	0.02	0.002	0.2	0.01	0.001	25.5	0.04
RD21	12	0.1	1.4	0.3	0.9	0.2	0.005	0.3	0.2	0.6	0.16	0.101	0.9	0.1	0.97	33.2	0.002	0.001	0.001	0.02	0.002	0.2	0.01	0.001	0.528	0.15
P-1A	50	0.1	1.5	0.1	0.5	2.1	0.005	0.8	4	200	0.53	5.29	2.9	0.1	1.02	33.7	0.007	0.012	0.007	0.04	0.002	0.2	1.8	0.001	8.87	0.34
P-5A	50	0.1	2.3	0.1	0.5	129	0.005	0.4	1.5	201	0.19	2.38	6.9	0.1	0.96	73.5	0.026	0.024	0.022	0.05	0.002	0.2	1.74	0.001	10.3	0.49
P-6A	52	0.1	1.9	0.3	0.5	33.1	0.005	1.1	1.8	196	0.39	9.84	8.6	0.1	0.88	18.5	0.047	0.084	0.066	0.04	0.003	0.2	11.9	0.001	37.7	0.88
P-9A	46	0.1	1.7	0.5	0.5	6.5	0.005	3	1.9	205	0.15	0.9	4.4	0.1	0.88	20.4	0.02	0.01	0.019	0.09	0.005	0.2	3.08	0.001	30.4	0.76
OS-13	19	<0.1	1.1	<1	<5	<1	<0.05	<3	<2	6	0.55	3.44	8.9	<1	<0.1	29.7	0.03	0.017	0.029	0.72	<0.02	<2	0.51	<0.01	152	0.38
OS-12	19	<0.1	2	<0.1	<0.5	2.1	<0.005	<0.3	2.4	20.4	0.6	3.1	8.5	<0.1	1.49	28.1	0.02	0.033	0.034	0.88	0.011	<0.2	31.3	<0.001	132	0.32
EMLD	--	--	--	--	50	50	--	20	2000	--	10	--	--	--	5	700 *	--	--	--	--	--	1	10	--	30 *	1.5

Values in µg/L, except F (mg/L). RD01 to RD21: alluvial groundwater, RD15: superficial water from Ridaura river, P-1A to P-9A: groundwater from granite border, OS-12 and OS-13: mineral water associated to granitic aquifer from the nearby Montseny range. EMLD: European maximum level for drinking water [4], *: WHO guideline value in drinking water [3].

**Table 5:** Factor loadings for the four first factors with Varimax normalized rotation. Results of sediments.

Variables	Factor	Factor	Factor	Factor
	1	2	3	4
**Hg**	0.86	−0.12	0.12	−0.11
**Au**	0.04	−0.13	0.07	0.10
**Cu**	0.80	−0.07	−0.11	−0.01
**Mo**	0.01	−0.01	−0.16	0.90
**Pb**	0.09	0.20	0.06	−0.08
**Ni**	0.94	−0.01	−0.05	0.15
**Zn**	0.46	0.17	−0.18	0.52
**As**	0.27	0.05	0.29	0.65
**Ba**	−0.06	0.52	−0.30	0.22
**Be**	−0.25	−0.26	0.81	0.13
**Co**	0.80	0.15	−0.23	0.37
**Cr**	0.91	0.04	−0.05	−0.007
**Li**	0.80	0.09	−0.16	0.31
**Mn**	0.28	0.13	−0.18	0.13
**Rb**	−0.13	−0.22	0.77	−0.19
**Sb**	0.59	−0.18	−0.04	−0.14
**Ti**	0.55	0.29	−0.31	0.49
**Th**	−0.07	0.96	0.11	−0.07
**U**	−0.13	0.33	0.72	−0.12
**V**	0.76	0.22	−0.28	0.45
**W**	0.12	0.72	0.10	0.12
**La**	−0.002	0.97	−0.16	0.02
**Ce**	0.005	0.98	−0.13	0.01
**Nd**	0.01	0.97	−0.12	0.04
**Sn**	−0.03	−0.06	0.15	−0.09

**Table 6:** Factor loadings for the four first factors with Varimax normalized rotation. Results of groundwater.

Variables	Factor	Factor	Factor	Factor
	1	2	3	4
**Li**	−0.05	0.18	−0.03	0.90
**Be**	0.96	0.02	0.06	0.01
**Ti**	0.99	0.01	0.06	0.01
**V**	0.99	0.03	0.03	0.02
**Cr**	0.97	0.03	0.05	−0.02
**Mn**	0.74	−0.19	−0.13	0.08
**Co**	0.98	−0.007	0.02	0.01
**Ni**	0.99	0.03	0.008	0.02
**Cu**	0.99	0.04	0.02	0.02
**Zn**	0.28	0.80	−0.09	0.22
**As**	0.96	−0.03	0.003	0.004
**Rb**	0.08	−0.04	−0.84	−0.04
**Mo**	−0.11	0.51	−0.73	0.22
**Sn**	0.96	0.005	0.04	0.05
**Sb**	−0.12	−0.72	−0.23	−0.07
**Ba**	0.76	−0.24	−0.28	0.12
**La**	0.98	0.03	0.05	0.01
**Ce**	0.99	0.04	0.02	0.01
**Nd**	0.97	0.03	0.05	0.01
**W**	0.23	0.41	−0.04	0.80
**Au**	−0.08	0.76	−0.04	0.16
**Hg**	−0.15	−0.48	−0.37	0.04
**Pb**	0.92	0.29	−0.05	0.01
**Th**	0.97	0.03	0.06	−0.02
**U**	−0.14	0.80	−0.22	0.11
**F**	−0.16	0.88	−0.08	0.04
